# Rare case of ocular tuberculosis in a diabetic patient: diagnostic and therapeutic dilemmas

**DOI:** 10.22336/rjo.2017.26

**Published:** 2017

**Authors:** Anca Pantalon, Emanuela Găman, Iustina-Silvia Crețu-Silivestru, Ciprian Danielescu

**Affiliations:** * “Sf. Spiridon” University Hospital, Iași, Romania; ** “Gr. T. Popa” University of Medicine and Pharmacy Iași, Romania

**Keywords:** ocular tuberculosis, Quantiferon, diabetes

## Abstract

We present the case of a patient who was diagnosed by chance with macular hypopyon during a conventional interdisciplinary examination. The clinical context and the association of a systemic disease, such as uncontrolled type 1 diabetes, rendered further investigations in this patient. Due to his immunocompromised status, etiology such as ocular fungi, lymphomas, tuberculosis was taken into account. Thorough complex investigations oriented the diagnosis towards ocular tuberculosis involvement.

## Introduction

Recent reports from the World Health Organization (WHO) mention that nearly two billion people, or one-third of the world’s population are infected by tuberculosis and that roughly 10% of the infected people are symptomatic [**1**]. Tuberculosis affects the lungs in 80% of the patients, while in the remaining 20% the disease may affect other organs, including the eye. Uveitis can be seen concurrently with tuberculosis, but a direct association is difficult to prove. Ocular tuberculosis is usually not associated with clinical evidence of pulmonary tuberculosis, as up to 60% of the extrapulmonary tuberculosis patients may not have pulmonary disease. The most frequently met ocular forms of TB are granulomatous chronic iridocyclitis, periphlebitis (Eales disease), choroiditis, or optic neuropathy associated with ethambutol.

The diagnosis of tuberculous uveitis is often problematic and in nearly all reported cases, the diagnosis was only presumptive. Tuberculous uveitis is a great mimicker of various uveitis entities and it can be considered in the differential diagnosis of any type of intraocular inflammation. It is still unknown if ocular manifestations result from a direct mycobacterium infection or hypersensitivity reaction and this is reflected in the management of tuberculous uveitis, which, if left untreated, inevitably leads to blindness [**2**]. The risk for tuberculosis in diabetic patients is greater than in the general population [**3**].

We present the clinical case of a 43-year-old male patient, who was referred to our clinic by the Diabetes and Metabolic Diseases Clinic for a routine fundus examination, without any subjective complaints (ocular or systemic). 

As personal history, in 2015, the patient was diagnosed with non-proliferative diabetic retinopathy – moderate form developed on the background of an uncontrolled diabetes firstly diagnosed in 1990. Systemic complications attributed to DM in this case were also diabetic nephropathy (stage III) and peripheral neuropathy, altogether with secondary arterial hypertension and hypercholesterolemia. Pulmonary tuberculosis was established as a diagnosis in 2005, but the patient did not follow the full therapy regimen and skipped most follow up visits in the Pneumology Clinic.

**Fig. 1:**
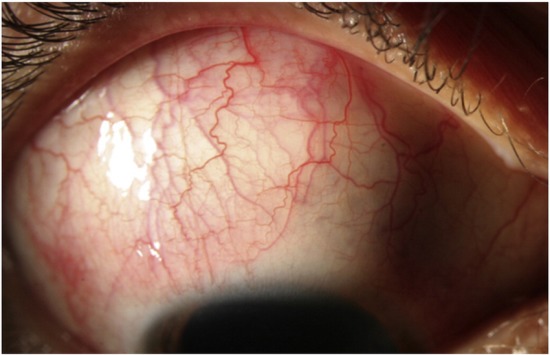
OD - Anterior segment examination: mild conjunctival congestion with episcleral vessels dilation

**Fig. 2:**
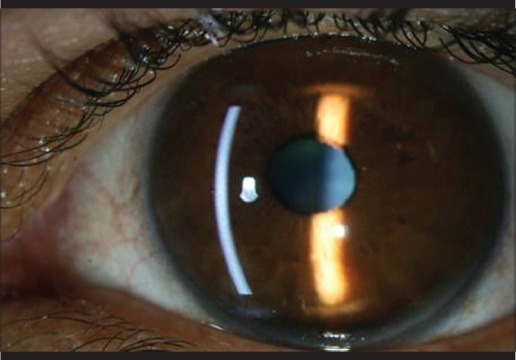
OS - Anterior segment examination: normal

The ophthalmological examination in January 2016 revealed a normal VA (1 nc, decimal scale) and good IOP in both eyes (13 mm Hg). The anterior segment examination can be followed in **Fig. 1**-**2** and revealed only a mild form of episcleritis (positive 1% Phenylephrine test). 

**Fig. 3:**
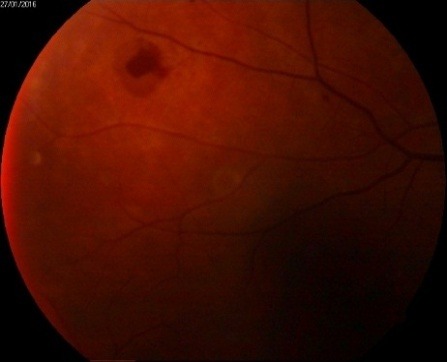
OD - Fundus examination (moderate non-proliferative diabetic retinopathy)

**Fig. 4:**
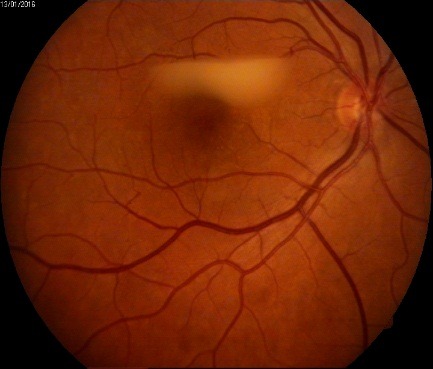
OD - Macular hypopyon

**Fig. 5:**
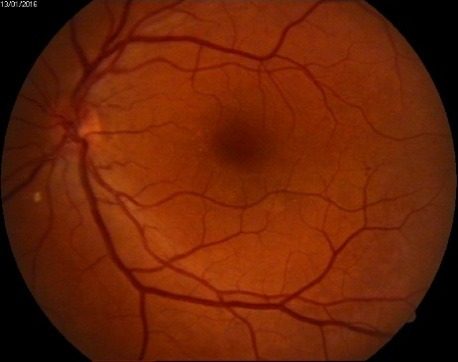
OS - Fundus examination (moderate non-proliferative diabetic retinopathy)

Fundus examination as revealed in **Fig. 3**-**5** proved the presence of microaneurysms, rare hard exudates in the central macular area in the OD and an intraretinal inkblot hemorrhage adjacent to the supero-temporal venous branch (**Fig. 3**), IRMA in the superonasal quadrant. Superior to the central macular region, a hypopyon was clearly visible (**Fig. 4**). Exudates were visible near the optic disk (< 1PD) in the OS cotton wool, few hard exudates in the macular area and IRMA in the nasal quadrant.

In this context and taking into account his general status (uncontrolled diabetes) and personal history of tuberculosis, we recommended pneumology reevaluation, QuantiFERON® testing and follow up visit in ophthalmology after 4 weeks. No treatment was administered either systemically or topically. 

At the later visit in February 2016, the aspect was stationary in both eyes, no sign of active intraocular inflammation was noticed, yet the positive Quantiferon® test (20 IU/ ml, normal range < 8 IU/ ml) alerted regarding a possible relapse of a TB infection with ocular involvement. Mantoux skin test was borderline positive (15mm/ 72h) at that time.

**Fig. 6:**
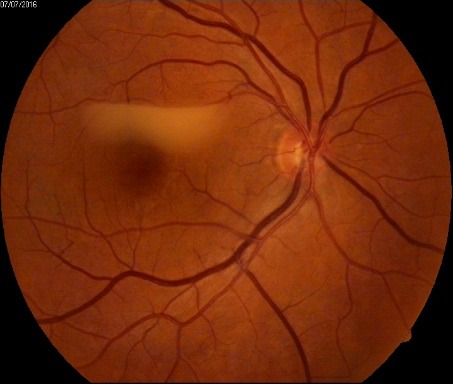
Macular hypopyon – stationary aspect at 6 months after presentation (no treatment was followed)

A thoracic X-ray and pneumology consult were part of the following assessment of this patient. Despite the stationary ocular lesions in the OD (**Fig. 6**), in July 2016, a full systemic exam revealed an impaired general state. 

**Fig. 7:**
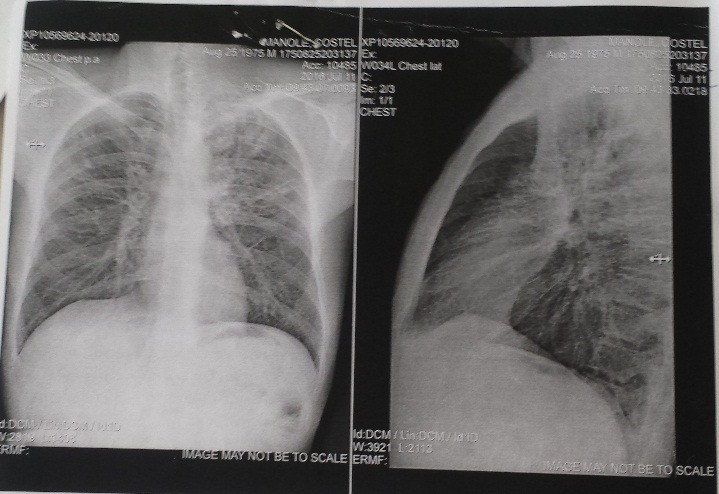
Thoracic X-ray: areas of non-homogenous condensation in both lungs, more prominent on the left side; bilateral hilar lymphadenopathy

Thus, marked weight loss (BMI = IMC = 19 kg/ m2), dyspnea, coughing were identified. The general exam revealed that the blood pressure was 130/ 80 mmHg, heart rate = 120 bpm and respiratory rate = 24 bpm. Crackles were present in the lower third of the left hemithorax. Despite the negative repeated sputum exams for Mycobacterium tuberculosis, thoracic X-ray (**Fig. 7**) pointed out a possible pulmonary TB reactivation in this patient, therefore a fiberoptic bronchoscopy with bronchoalveolar lavage and trans-bronchial biopsies was performed on the lower left lobe. 

Direct exams for regular bacteria, fungi in the bronchoalveolar lavage were negative; optic microscopy (acid-fast bacilli) was positive for Mycobacterium tuberculosis. Culture from the bronchoalveolar lavage also showed the presence of Mycobacterium tuberculosis. 

**Fig. 8:**
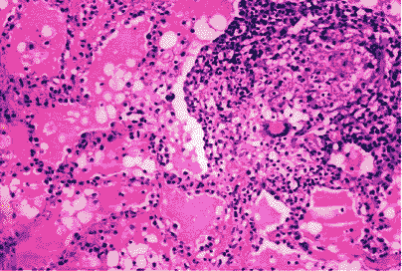
Lung biopsy (HE coloring)

**Fig. 9:**
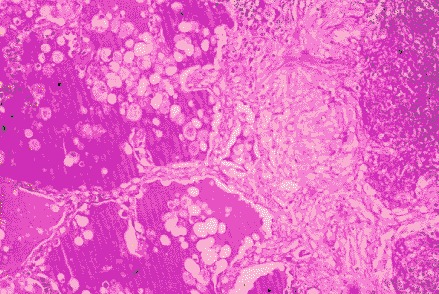
Lung biopsy (PAS coloring)

The trans-bronchial biopsies showed non-specific inflammatory changes. In hematoxylin eosin coloring (**Fig. 8**), the lung tissue fragment contained multiple granulomatous lesions and caseous necrosis, epithelioid histiocytes and giant multinucleous cells, rare lymphocytes. The alveoli space was occupied by acidophilus material and activated macrophages. There was an intense staining of the material present within the pulmonary alveoli (PAS+) in PAS coloring (**Fig. 9**). Due to changes in histology, a supposition of alveolar proteinosis was raised in this case, in addition to the pulmonary tuberculosis.

Laboratory analysis showed anemia, normal renal function, poor glycemic control, inflammatory changes and anti-infectious leukocyte response, ventilatory dysfunction with altered oxygen saturation. No other parameters were modified, nor were other infectious causes found (including negative HIV serology). 

The patient’s parameters were WBC count = 12,200/ mm3; RBC count = 3.67 million/ mm3; ESR = 29.4%; Hemoglobin = 10g%; Platelets = 545,000/ mm3; Glucose = 223 mg/ dL, Urea = 22mg/ dL. Creatinine = 0.8mg/ dL, Na = 136mEq/ L; K = 4,7mEq/ L; Ca = 9.7mg/ dL, Mg = 1.6mg/ dL, AST: 44 UI/ L; ALT: 13 UI/ L, Total bilirubin: 0.4 mg/ dl (direct: 0.1 mg/ dl), Prothrombin time: 68% (13.3" INR: 1.24). APTT: 32", ABG's (FI02 = 0,21): pH: 7.4; PaO2:76.5mmHg; PaCO2: 40.4mmHg; HCO3 = 25.1mEq/ L; SatO2 = 95.3%.

According to WHO tuberculosis treatment guidelines [**1**], a standard anti-TB treatment (ATT) was planned. Thus, in the first 8 weeks, the patient was recommended Rifampin, Isoniazid, Ethambutol, and Pyrazinamide, then for the next 18 weeks a regimen with: Rifampin and Isoniazid should have been followed. Ambulatory follow up was recommended after the first 2 months. The last visit showed no change in the hypopyon appearance, a fact that pleaded more for a local delayed hypersensitivity reaction than for an acute intraocular infection, which should have responded to the antimicrobial treatment.

## Discussions

The WHO reported an increasing number of TB infections in both the low/ middle-income and high-income countries due to multidrug-resistant TB, HIV and global migration [**1**]. This globalization and resurgence of TB means that ophthalmologists now see a spectrum of diseases: from eyes with obvious clinical OTB to mildly symptomatic patients with uveitis associated with an occult TB infection [**4**].

The term “ocular TB” describes an infection with the M. tuberculosis species that can affect any part of the eye (intraocular, superficial, or surrounding the eye), with or without systemic involvement. “Secondary ocular TB” is defined as an ocular involvement as a result of seeding by hematogenous spread from a distant site or direct invasion by contiguous spread from adjacent structures.

Intraocular tuberculosis (TB) is rare and represents a great mimicker of various clinical entities, therefore should be considered in the differential diagnosis of any ocular inflammatory condition. Primary ocular localization is rare (direct inoculation); therefore, cases that are more frequent occur via secondary hematogenous dissemination from remote sites (lungs, most frequently). Ocular involvement in tuberculosis signals an active infection; still, some cases represent only remote immune reactions (delayed hypersensitivization type IV). Ocular inflammation could be unilateral or bilateral; sometimes inflammation of one eye starts months or years before the other [**4**]. Forms of uveitis can reach the anterior segment, intermediate or posterior segment or all together (panuveitis).

Posterior uveitis is the most common presentation of intraocular TB, with lesions predominantly present in the choroid, such as focal, multifocal or serpiginous choroiditis, solitary or multiple choroidal nodules (tubercles), choroidal granuloma (tuberculoma), neuroretinitis, subretinal abscess, endophthalmitis, panophthalmitis, and retinal vasculitis, which is frequently ischemic in nature, and may lead to proliferative vascular retinopathy with recurrent vitreous hemorrhage, rubeosis iridis, and neovascular glaucoma [**5**]. The retina can indirectly be involved with ocular tuberculosis due to an associated choroiditis, but direct retinal involvement is rare [**6**]. In the event of primary retinal involvement, the presentation is either as a presumed tubercular retinal vasculitis or as Eales’ disease, an associated vasculitis thought to represent a hypersensitivity reaction to tuberculosis [**7**]. Vitritis, retinal hemorrhages, and neovascularization are also regularly associated [**6**]. Periphlebitis is often present in Eales’ disease as well, but there is no other evidence of intraocular inflammation [**7**]. 

Our patient manifested none of the above-mentioned clinical features. Moreover, he exhibited an excellent visual acuity that was maintained during the 12 months follow up interval, therefore, we considered it unnecessary to perform any supplementary investigations (OCT exam, fluorescein angiography, etc.) to document potential changes in the posterior segment. Anyhow, the clinical findings mentioned above are suggestive, but nonspecific and it is still unknown if ocular manifestations result from a direct mycobacterial infection or a hypersensitivity response to mycobacteria [**8**]; this being reflected on the management of TB uveitis. 

Moreover, due to the Jarisch-Herzheimer reaction, after the initiation of any antituberculostatic treatment, some patients manifest exacerbation of the intraocular inflammation, but in our case, the eye remained silent after 2 months of ATT. Yet this lack of response could be attributed to the general immune status of the patient. This also supports the hypothesis that the appearance of the macular hypopyon was based on a hypersensitivity reaction and not a real intraocular infection/ inflammation due to MBT localization.

In most studies, the diagnostic criteria for presumed ocular tuberculosis were: residence or migration from the areas endemic in TB, previous history of contact with TB-infected patients, presence of suggestive ocular findings, exclusion of other known causes of ocular inflammation, corroborative evidence such as a positive TST (tuberculin skin test), positive interferon-gamma release assays (QuantiFERON), and a positive response to conventional ATT without recurrence. An extraocular evidence of TB in a patient with ocular inflammation also aids in diagnosing intraocular TB [**9**].

Especially when poorly controlled, the functional damage to the polymorphonuclear neutrophils (adherence, chemotaxis and bactericidal activity), together with a dysfunction of the monocyte macrophage system and of cellular immunity, determine a greater risk of infections or impaired immune response in diabetic individuals [**3**]. In these individuals, the risk for tuberculosis is 2.0 to 3.6 times greater than in the general population [**3**].

Quantiferon tests (IGRA) are based on gamma interferon production by T cells sensitized to specific antigens, which are specific to MTB and therefore are not influenced by BCG and most nontuberculous bacteria. IGRAs are more specific and sensitive than PPD tests in detecting active pulmonary TB infections [**10**]. However, they are less sensitive in diagnosing latent TB infections. Also, Quantiferon is more specific in diagnosing TB-associated uveitis, and serves as a better diagnostic tool if used in conjunction with the TST [**11**]. The accuracy of diagnosing TB uveitis increases when both tests are used in combination with suggestive clinical signs [**11**]. 

Based on the entire assessment of this patient we chose not to treat OTB with any topical medication, since the general treatment follows the same guidelines as for active pulmonary and extrapulmonary and was recommended as it follows - first 8 weeks Rifampin, Isoniazid, Ethambutol and Pyrazinamide, then the next 18 weeks Rifampin and Isoniazid. An escalation of corticosteroids may have been helpful in case of increased ocular inflammation during treatment, but it was not the case of our patient. On the other hand, response to treatment in OTB is slow, therefore at 2 months follow up the stationary aspect of the “macular hypopyon”. We have used this term “macular hypopyon” to describe what appeared to be an accumulation of white material in front of the macula with a horizontal level (fluid-like). The term has been borrowed from the description of an endophtalmitis, where it describes an accumulation of pus on the macula due to the supine position [**12**] similar to what we found in our patient, but we could not identify exactly the composition in the pre-retinal deposit. Therefore a differential diagnosis should also take into account a preretinal hemorrhage where the blood has degraded and took this aspect.

A particularity of this case was the incidental manner of discovering a relapse of pulmonary TB via a routine ophthalmological fundus examination. A corroboration of personal history, clinical aspect, and systemic status oriented the diagnosis of ocular TB. Modern laboratory measurements (Quantiferon), combined with old standard tests (TST) aided a more accurate, yet still not certain OTB diagnosis. However, the detection of acid-fast bacilli (AFB) with Ziehl-Neelsen or auramine rhodamine stains has a low yield even from the aqueous or vitreous [**6**], cultures are time-consuming (6-8 weeks) and other techniques (e.g. PCR), which attempt to isolate MTB from the ocular samples, may be challenged by the low sample volume and the paucibacillary nature of the disease. Alternatively, the poor positive outcome of these methods may be explained by the second hypothesis of OTB, being an immune-mediated mechanism of inflammation.

Therefore, obtaining histopathologic evidence is preferable, but difficult to obtain in adequate size to support the diagnosis of ocular tuberculosis. In this case, the typical histology aspect was obtained only from the pulmonary biopsy. Altogether, this exam also detected another incidental association in this patient (alveolar proteinosis) which could confound the thoracic X-ray interpretation in a tuberculosis case. The incidence of PAP in the general population is 1 in 2 million people [**13**], with a 3 to 1 male-to-female predominance. Almost 80% of the patients are between 20 and 50 years old, although it has also been described in newborns and school age children [**14**] and in the elderly [**15**]. The main symptom is exertion dyspnea. Many patients present with dry cough or with opalescent and viscous sputum. Asthenia and weight loss may be present and these could also be confounders for our patient. Fever is more common in the secondary form and was not the situation here. Chest pain and hemoptysis are not frequent in the primary form. The physical examination is nonspecific, with predomination of crackles in the affected areas [**13**]. The most common X-ray pattern is a symmetrical, bilateral, alveolar infiltrate, predominantly in the lower lobes, as it was the case in this patient.

Further follow up is needed as relapses are silent and frequent in immunocompromised patients, therefore a multidisciplinary approach is required to further manage this case.

## Conclusion

We presented a case of presumed ocular tuberculosis with a single non-specific lesion (macular hypopyon) and no active ocular inflammation associated with it, in a diabetic young patient. Recent advances in diagnostic tools for OTB immunological techniques (Quantiferon) have improved the specificity of making this diagnosis. However, the clinical diagnosis of OTB remains a complex issue, as these investigations are mainly adjunctive and complementary, while the clinical manifestations of OTB are highly variable.
